# Non-thermal Electroporation Ablation of Epileptogenic Zones Stops Seizures in Mice While Providing Reduced Vascular Damage and Accelerated Tissue Recovery

**DOI:** 10.3389/fnbeh.2021.774999

**Published:** 2021-12-24

**Authors:** Emma Acerbo, Sawssan Safieddine, Pascal Weber, Boris Botzanowski, Florian Missey, Marcel Carrère, Robert E. Gross, Fabrice Bartolomei, Romain Carron, Viktor Jirsa, Ivo Vanzetta, Agnès Trébuchon, Adam Williamson

**Affiliations:** ^1^Institut de Neurosciences des Systèmes, UMR 1106, Aix-Marseille Université, Marseille, France; ^2^Department of Neurosurgery, Emory University School of Medicine, Atlanta, GA, United States; ^3^Department of Functional and Stereotactic Neurosurgery, Timone University Hospital, Aix-Marseille Université, Marseille, France; ^4^Institut de Neurosciences de la Timone, CNRS, Aix-Marseille Université, Marseille, France; ^5^Center for Bioelectronic Medicine, Department of Medicine, Solna, Karolinska Institutet, Stockholm, Sweden

**Keywords:** resective surgery, ablation, two photon, epilepsy, electroporation

## Abstract

In epilepsy, the most frequent surgical procedure is the resection of brain tissue in the temporal lobe, with seizure-free outcomes in approximately two-thirds of cases. However, consequences of surgery can vary strongly depending on the brain region targeted for removal, as surgical morbidity and collateral damage can lead to significant complications, particularly when bleeding and swelling are located near delicate functional cortical regions. Although focal thermal ablations are well-explored in epilepsy as a minimally invasive approach, hemorrhage and edema can be a consequence as the blood-brain barrier is still disrupted. Non-thermal irreversible electroporation (NTIRE), common in many other medical tissue ablations outside the brain, is a relatively unexplored method for the ablation of neural tissue, and has never been reported as a means for ablation of brain tissue in the context of epilepsy. Here, we present a detailed visualization of non-thermal ablation of neural tissue in mice and report that NTIRE successfully ablates epileptic foci in mice, resulting in seizure-freedom, while causing significantly less hemorrhage and edema compared to conventional thermal ablation. The NTIRE approach to ablation preserves the blood-brain barrier while pathological circuits in the same region are destroyed. Additionally, we see the reinnervation of fibers into ablated brain regions from neighboring areas as early as day 3 after ablation. Our evidence demonstrates that NTIRE could be utilized as a precise tool for the ablation of surgically challenging epileptogenic zones in patients where the risk of complications and hemorrhage is high, allowing not only reduced tissue damage but potentially accelerated recovery as vessels and extracellular matrix remain intact at the point of ablation.

## Introduction

The primary method of treatment for patients suffering from drug-resistant focal-onset epilepsy is resective surgery (Wiebe et al., 2001). In the most common type of drug-resistant epilepsy, mesial temporal lobe epilepsy (MTLE), while associated with high-rates of seizure freedom surgical resection can often impact neurocognitive function. For drug-resistant MTLE, focal thermal ablation is a less-invasive method being explored for surgical treatment to achieve high-degrees of seizure freedom while maintaining memory and other cognitive functions associated with the basal and lateral temporal areas (Bezchlibnyk et al., 2018; Gross et al., 2018). Although, radio-frequency (RF) ablation and laser interstitial thermal therapy (LITT, aka laser ablation) are the methods with the most attention, achieving reasonable seizure-free rates with less negative impact on neurocognitive function, (Gross et al., 2015) there remain technical questions about these two methods of thermal ablation, primarily thermal injury to surrounding tissues, edema due to hemorrhage (Howenstein and Sato, 2010) and poor directionality.

Here we investigate irreversible electroporation, a common method of focal ablation outside of the brain, but which may be feasible to use in brain tissue to avoid the primary concerns of focal RF and laser ablation in epilepsy. Indeed, in spite of the numerous focal ablations performed using irreversible electroporation in tumor removal, (Davalos et al., 2005; Golberg and Yarmush, 2013) it has not yet been adopted in the ablation of neural tissue clinically. The NTIRE mechanism itself is well-understood. Electroporation induces the formation of pores in the cell membrane due to the application of an electric field with a relatively high field intensity (typically in the kV/cm range), however, with a very short pulse duration (in the μs range) to avoid thermal heating (Kinosita and Tsong, 1977). Classically, electroporation was a technique used to insert plasmids into mammalian cells (Chu et al., 1987). Here, we visualize and investigate the possibility of using non-thermal irreversible electroporation (NTIRE) in the somatosensory cortex, posterior parietal cortex, and hippocampus of mice. For this purpose, we induce pores in the target neurons to be ablated, allowing the influx and efflux of ions and molecules, irreversibly disturbing the intra- and extracellular concentration gradients. The target neurons are not able to recover from these abrupt, non-thermally induced physiological changes, and subsequently die (Joshi and Schoenbach, 2002). There are many studies showing the negative impact of elevated temperatures on tissue, creating necrosis and tissue damage past a certain threshold (Moritz, 1947; Yarmolenko et al., 2011). Here, in applying multiple temporally short pulses of electric fields, it’s possible to create irreversible electroporation without heat-related damage (Joshi and Schoenbach, 2002).

We found that neural cells can be ablated in specific regions while the blood-brain barrier remains intact in the same region. Vasoconstriction and leaking from the blood-brain barrier during and after the NTIRE event is investigated as a function of vessel size, with larger vessels essentially temporarily contracting after the electroporation without any significant degree of leakage and small vessels temporarily contracting with only a mild degree of leakage. However, both vessel groups remain intact, while target neurons around the vessels are destroyed. Additionally, re-innervation of fibers into ablated regions and activation of dendritic cells indicates a possible acceleration of recovery in the ablated brain region compared to a thermal ablation where coagulated tissue renders a brain region permanently dead.

## Materials and Methods

### Surgeries and Electrodes Placement

All surgical procedures were approved by the National Animal Studies Committee of France (authorization no. APAFIS20359-2019041816357133v5). We used 27 OF1 (Charles Rivers, Les Oncins, France) adult mice 8–10 weeks old, 30–40 g in weight. We used also 6 mice with Thy1-cyan fluorescent protein (CFP)-23, LysM-green fluorescent protein (GFP), CD11c-yellow fluorescent protein (YFP). Mice were deeply anesthetized with ketamine–xylazine (120 mg/kg, 12 mg/kg, respectively) in saline with (IP) intra-peritoneal injection and supplemented when need it (60 mg/kg, 6 mg/kg). Ocry-gel (Laboratoire TVM, Lempdes, France) was applied to maintain eye moisture and surgical anesthesia was verified by a lack of toe pinch reflex. The hair around the surgical site was shaved, and the skin was cleaned with iodine solution (Vétoquinol). The skin of the skull was incised, and the fascia was gently removed using a scalpel.

For the placement electrode on the cortex, a U-shaped clip were placed on the skull of the left side and glued with primer dental cement (GACD, France) followed by blockout resin photopolymerized by an optic UV lamp (GACD, France). This clip served to fix mice during surgery procedure and image acquisition under the microscope, to a holder. A craniotomy was carried out on the right hemisphere of the head using a high-speed drill with a carbide bur (size = round 1/4; World Precision Instruments, Hitchin, England). The brain was kept free of debris by repeated rinsing with phosphate buffered saline (PBS) during implant and surgery.

Two 50 μm platinum-iridium microelectrodes (Phymep) were placed on the dura matter of the brain and fixed on the skull by primer dental cement followed by the photopolymerized block resin. Prior to the surgery, a glass window of 5 mm diameter and 0.17 mm thickness (Thermo Fisher Scientific, France) was cleaned and dried. Once the electrodes were positioned, the glass window was placed over the skull and electrodes. Gentle pressure was applied to maintain the glass in place while dental cement was applied to the edges of the glass and the surrounding bone and muscle. Finally, an additional layer of dental cement was applied over the primer to reinforce the structure.

For the deep brain ablation, groups of OF1 underwent a surgical procedure as described above. However, instead of placing **two** wires on the cortex, an implantable electrode was placed into the brain in order to reach the hippocampus. For that, a craniotomy was performed by drilling a hole for the electrode (PT electrode twisted 5 mm, Bilaney Consultant**s**, Germany) (Coordinates: ML: −2.04, AP: +2.07, DV: 1.29 determined by the Paxinos atlas) and another one for the reference in the cerebellum. At the end, electrodes were cemented with Dentalon (PHYMEP, France).

### Non-thermal Irreversible Electroporation and Thermal Irreversible Electroporation Lesion

The electroporator device (Electro cell B10-CNRS) is connected through the two wires placed on the brain of the mice. The delivered voltage values depend on the space between the two wires, but it was always equal to 1,750 V/cm. The width of the pulse for both the positive and negative phase was 50 μs the frequency was 10 Hz with 10 repetitions. All TIRE and NTIRE stimulations were performed with the electroporator device as it is possible to configure parameters of stimulation (Supplementary Figure 1).

### Microscopic Imaging

For the microscopy, animals were injected with 50 μl of Dextran, Rhodamine B, 10,000 MW, Neutral (Thermo Fisher Scientific) (3 mg/ml) in saline was injected in the ophthalmic venous sinus using the retro-orbital method. During imaging, the animals were freely breathing, and the microscope chamber was heated to ∼32°C. We used a tunable femtosecond pulsed laser (Mai-Tai, Spectra Physics, Évry, France) coupled to a Zeiss two-photon microscope (LSM 780) equipped with a Plan-Apochromat 20× water immersion objective lens (NA = 1.0) and five non-descanned detectors (NDD). For each image stack laser, intensity and sometimes the gain, were adjusted according to imaging depth in order to maximize image intensity while minimizing saturation throughout the image stack. We always imaged the region between the two wires using the following strategy. The window was epi-illuminated with LED lights on the side of the objective. To determine the vasoconstriction percentage, blood vessels where divided into two groups: large blood vessel ≥20 μm and small blood vessel <20 μm. Images were analyzed using the ZEN 2.6 software, the diameter of blood vessels before and after stimulation was taken, and the percentage of vasoconstriction was calculated.

### Kindling Protocol

For depth NTIRE and TIRE ablation of epileptic foci, mice with implantable hippocampal -CA3- electrode (Twisted Pair, Plastic One^®^), after 2 days post-surgery, were connected to a data acquisition system (Intan Technologies, United States) in order to record and stimulate. After finding the after discharge (AD) threshold for each mouse (the minimum current applied to evoke a seizure) the following parameters were applied: 50 Hz, 500 **μ** s pulse width, train of 500 stimulation pulses, with current set at the AD threshold (see Supplementary Figure 2 for more details). This stimulation was repeated 10 times every 10 min (Lothman et al., 1985; Musto et al., 2009). After that, NTIRE or TIRE ablation was performed with the electroporator device (under anesthesia) and epilep**ti**form events were recorded within the following 12 h.

### Analysis Spikes Rate

To detect specific events as spikes, we performed a semi-automatic detection on the signal. The detection part was processed by using Delphos software, a detector of spikes and oscillations used mainly in clinical research (Roehri et al., 2018). Before to analyzed the signal, it has been downsample to 3,750 Hz and filtered in between 20 and 40 Hz. All the spikes detection were reviewed by a single person (EA) via AnyWave software, a visualization software for electrophysiological data (Colombet et al., 2015). To asses the number of epileptic spikes, each mice underwent a 1 h recording before NTIRE treatment and 1 h after the treatment (for non-treatment group, delay in between the two recording session was the same as NTIRE group). Epileptic spikes were detected and an average of the spikes/min was calculated.

### Finite Element Method

Finite Element Method calculations were performed with the COMSOL Multiphysics 5.5 software. The physic used was “Electrical Current.” A custom mouse brain mesh model was designed on Blender software and then imported in COMSOL Multiphysics 5.5. The distance and the voltage applied between the electrodes were taken in account to recreate as closely as possible the experimental conditions of the Cortical NTIRE ablation, 245 V between ≃ 1.4 mm. All plots were made and exported with the built-in tool of COMSOL Multiphysics 5.5 (Supplementary Figure 3).

### Histology

Conventional histology was performed on twelve section. Four days after the stimulation mice were perfused with saline followed by 4% paraformaldehyde, then brain was carefully removed from the skull and transferred to 4% PFA for post fixation. After 1 day of post-fixation, brain was washed three times in 0.12 PB and then immersed in 20% sucrose in 0.12 PB for 24 h. The day after brain was frozen, with the ventral surface placed on a metal plate, cooled by a surrounding bath of dry ice. Brains were stored at −80°C until cut. The brain tissue was sectioned into coronal section of 30 μm in thickness on Leica CM3500 cryostat (Leica Microsystems Inc., Bannockburn, IL, United States).

For cell counting: all tissue sections lying between the two electrodes were collected. Brain sections to be stained with Nissl Eosin were directly mounted on the microscopic glasses (Polysine™ Microscope Adhesion Slides – Thermo Fisher Scientific) and left for 24 h in the oven.

Then, sections were placed in alcohol 95% two times for 10 min, followed by 5 min in chloroform. Then, they were hydrated by alcohol 95, 80, and 75% for 2 min each. Next, sections were soaked 10 times slowly in distilled water before staining them in the heated (60°C) Nissl’s solution for 1 min. A second wash with water is done for 1 min before immersion of the sections in acetic formalin solution for 6 min. Washing with water for 1 min is done before passing to the dehydration step in alcohol 80, 90, 95, 100, and 100% for 2 min each. Nissl’s sections were used for assessing the cell death.

### Statistics

Date were analyzed using R software. Vasoconstriction, cell loss and spike rate data were separated into groups regarding the ablation protocol (NTIRE, TIRE, and no treatment). After a common normality verification using Shapiro test, groups were compared with *T*-test or Wilcoxon-test depending on the presence or absence of a normal distribution, respectively.

Figures 2, 3: 9 NTIRE mice were analyzed to assess the percentage of vasoconstriction on NTIRE mice. To determine the vasoconstriction percentage broadly, blood vessels where divided into two groups: large blood vessels ≥20 μm and small blood vessels <20 μm.

Figure 4: 4 OF1 mice were sacrificed, and histological analysis of cell loss was performed for cortex cell-density data, with contralateral cortex used as the control for cell density.

Figure 5: 12 OF1 mice were kindled as described above and six received NTIRE ablations, the six other were not treated to show that there is no decrease of spikes during time. After a common normality verification using Shapiro test, Wilcoxon test were performed.

## Results

### Experimental Set Up

**FIGURE 1:**
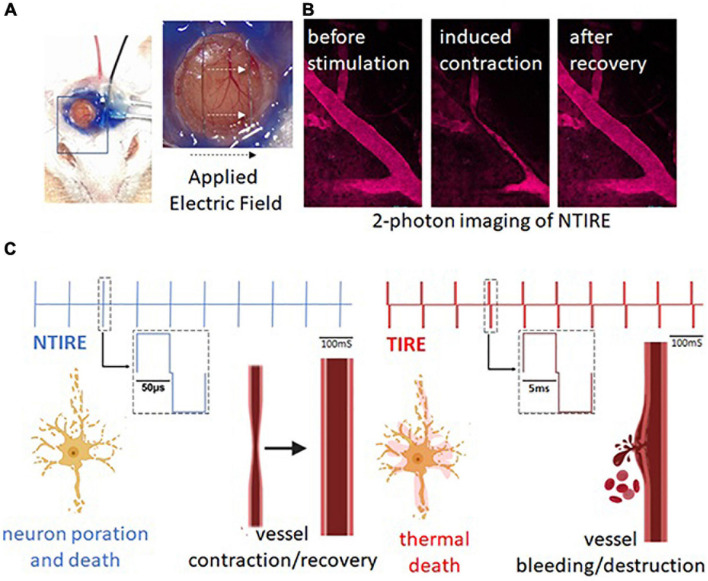
Visualizing vessel contraction and the concept of NTIRE to ablate epileptic foci. **(A)** Mice are implanted with microelectrodes and cranial windows. In the simplest configuration, the electric field for NTIRE or TIRE is applied between the two microelectrodes under the cranial window (white arrows). **(B)** 2-photon fluorescence imaging is used to visualize the contraction or destruction of blood vessels due to the applied electric field. **(C)** The primary difference between NTIRE (non-thermal) and TIRE (thermal) ablation process is the duty cycle of the stimulation. NTIRE uses very short pulses per period at high-voltage which ablate neurons non-thermally via electroporation while vessels remain in place. TIRE uses longer pulses per period which coagulate tissue, ablating neurons thermally and causing destruction of vessels and bleeding (detailed discussion of NTIRE and thermal versus non-thermal ablation parameters in Supplementary Figure 1).

**FIGURE 2:**
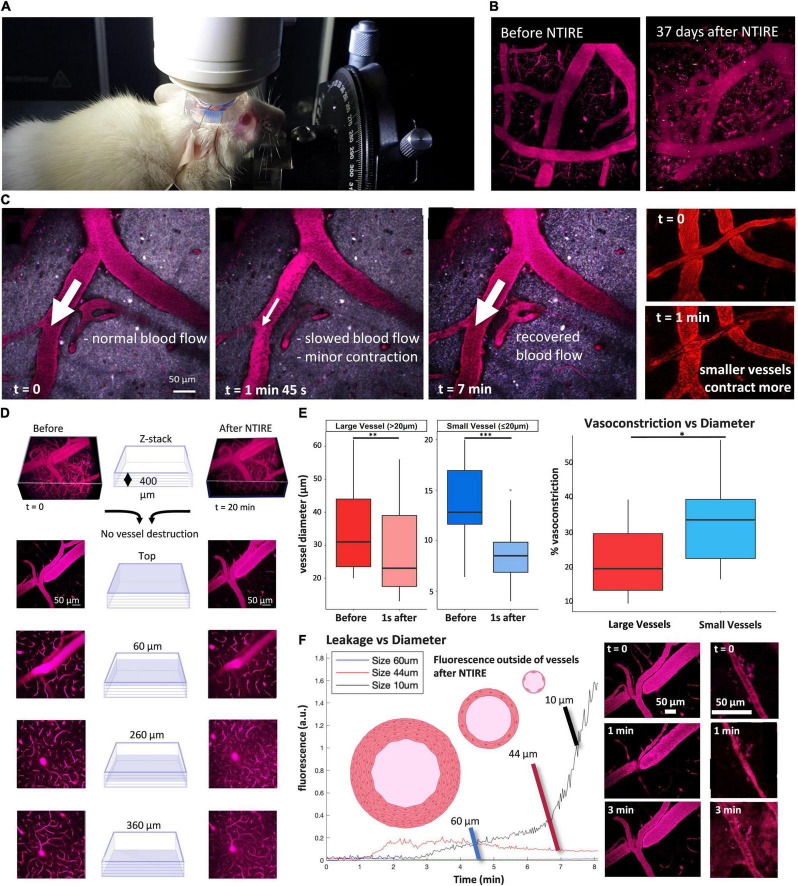
Principal of NTIRE: Contraction, Recovery, and Leakage. **(A,B)** Mice and blood vessel integrity were monitored for a maximum of 37 days after the NTIRE process, with no discernable loss of vessels in the time window. **(C)** Blood flow (represented by the white arrows) is normal, then slowed in the minutes after the NTIRE event, and recovers for all vessels by 7 min. **(D)** 400 μm × 400 μm block of cortex taken between the two microelectrodes (schematically seen in Figure 1). Imaging immediately before and 7 min after NTIRE (1,750 V/cm, 10 Hz) reveals no obvious destruction of vessels, independent of vessel size. **(E) Contraction:** The average contraction of vessels due to NTIRE is measured, for vessels above vs. bellow 20 μm diameter, before and 1 s after NTIRE [Large vessels: *p* value (Shapiro test) >0.05, *p*-value (paired *t*-test) = 0.0003741. Small vessels: *p*-value (Shapiro test) <0.05, *p*-value (paired Wilcoxon test) = 0.001953]. Recovery takes approximately 1 min. The percentage of vasoconstriction for smaller vessels (diameter less than 20 μm) approaches 50% [*p* value (Shapiro test) >0.05, *p*-value (paired *t*-test) = 0.02622]. **(F) Leakage:** Leakage from vessels is estimated by considering the increase in fluorescence at a distance of 10 μm from an individual vessel. In the example here, the leakage from smaller vessels (diameter less than 20 μm – seen in the example panels, bottom right) continues for many minutes and is greater than from larger vessels, where the largest vessels (+60 μm) simply show no leak.

The experimental setup is introduced in Figures 1A,B, where microelectrodes are mounted on the somatosensory or posterior parietal cortex of mice. When the NTIRE event is visualized in real-time using a 2-photon fluorescence microscope, a brief contraction of vessels can be seen but they remain intact. However, neurons in the region of the NTIRE are destroyed, as depicted in Figure 1C. A thermal ablation, or thermal irreversible electroporation (TIRE) also uses a high electric field, but typically the pulse width is larger creating a thermal event and subsequent coagulation necrosis of tissue – vessels, neurons, and all tissues in the ablated region are destroyed. Then we wanted to compare if the tissue damage in the epileptic hippocampus after NTIRE ablations is reduced compared to epileptic mice receiving a thermal ablation of the same region. To better understand this, the parameters of non-thermal ablations are discussed in detail in Figure 2 and Supplementary Figure 1.

### Non-thermal Irreversible Electroporation Induces Vessels Contraction Without Ablation

**FIGURE 3:**
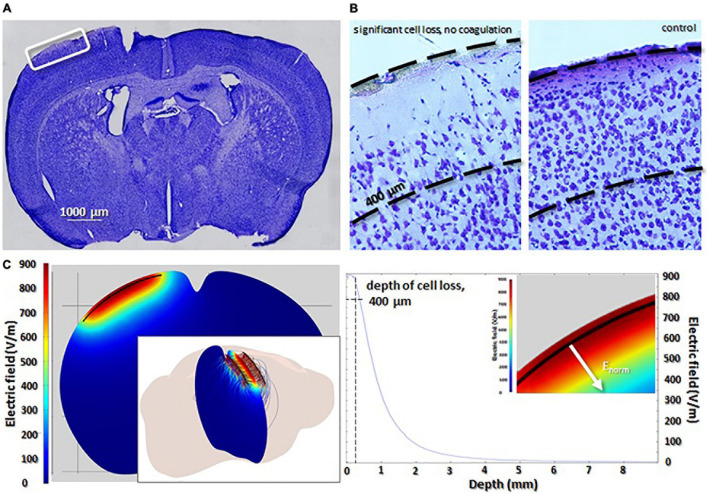
Ablation of neurons using NTIRE. **(A)** Coronal section highlighting the location of microelectrodes on the cortex. **(B)** A magnified view of the highlighted region in **(A)**. Significant cell loss is seen down to a depth of approximately 400 μm. Control sections (left panel) highlight the density of cells without the NTIRE ablation. **(C)** Finite-element method simulations representing the penetration of the electric field into the tissue during one pulse of the NTIRE ablation (detailed discussion of NTIRE pulses in Supplementary Figure 1). The depth of the electric field perpendicular to the microelectrodes. Clearly, the depth of cell loss at approximately 400 μm corresponds to an electric field of 800 V/cm. Values of electric field below this number seem to not cause NTIRE ablation. In the inset, absorption of the electric field is shown in the cortex.

The primary parameters of interest in a thermal versus non-thermal ablation are power and electric field. The electric field must be sufficiently high to cause the irreversible poration of cell membranes. An ablation is performed with identical electric field values, high enough to cause irreversible poration, here 1,750 V/cm (Supplementary Figure 1). However, in NTIRE ablation, the associated power is only 200 mW, creating a non-thermal event. As can be seen in the image, the cortex immediately after the NTIRE event is visibly unchanged. For TIRE, the associated power is 11 W, creating a very thermal event. As can be seen in the image, the cortex immediately after the TIRE event shows a increase in bleeding. Because of absence of bleeding in the NTIRE process, mice can be monitored for longer periods of time without significant reduction in imaging quality of the two photon visualization. Consequently, mice were monitored up to 37 days after NTIRE ablation, with no visual destruction of vessels seen for the duration of the monitoring (Figure 2B). As seen in Figure 2C, blood plasma flow slows, with red blood cells seen individually in the vessels (panel, *t* = 1 min 45 s) as the speed of blood plasma flow has decreased. Flow recovers for vessels of all sizes after several minutes, however, (right panel) the difference between large and small vessels is simply the percentage of contraction – with no discernable long-term effects. Indeed, it was the short-term effects on vessels which were quite interesting immediately after NTIRE, namely the leakage and the percentage of contraction. Then, the contraction and leakage as been analyzed as a function of diameter. In Figure 2D, a section of cortex can be seen with vessels down to a depth of approximately 400 μm. Vessels of numerous sizes can be seen before and after, without any damage to the vessels. Vessel diameters due to the NTIRE have completely recovered. On the panel E, data showing typical contractions in a section of tissue is grouped into large vessels (greater than 20 μm diameter) and small vessels (less than 20 μm diameter). The percentage of vasocontraction is more dramatic for smaller vessels than larger vessels (*t_test: p*-value = 0.02622). Interestingly, this corresponds well to leakage, which is measured as a change in fluorescence measured 10 μm outside of vessels. So, three example vessels are shown, with 60, 44, and 10 μm diameters. The change in fluorescence outside of all vessels of 60 μm in diameter or greater is negligible, which is an important result as damage and leaking to such large vessels would create a significant hemorrhage. The change in fluorescence outside of vessels of large-medium vessels 30–40 μm in diameter (40 μm in the example) is small and relatively fast (Figure 2F). The interesting measurements are from vessels less than 20 μm, (10 μm diameter in the example) where fluorescence increases substantially. Although the vessels are not destroyed, leakage will likely allow a small amount of blood into the surrounding tissue. The result is that leakage from a vessel due to NTIRE seems to scale with the percentage of vasoconstriction of a given vessel, with an increased percentage in vasoconstriction from smaller vessels correlating with increased leakage. However, independent of size, vessels remain intact over time with contraction and leakage limited to the initial time immediately after NTIRE. In contrast, the consequences for neurons due to NTIRE is not temporary, as seen in Figures 3A,B and Supplementary Figure 4.

### Non-thermal Irreversible Electroporation Can Ablate Neurons

As depicted in Figure 3, ablation of neurons scales very closely with the electric field. In Figure 3A a section of cortex directly between the two micro electrodes can be seen. Figure 3B shows a loss of neurons can be seen in the region above 400 μm which, as shown in Figure 3C, correspond to a field strength of ∼800 V/cm; this correlates well with the threshold for irreversible electroporation. The electric field below the depth of 400 μm is not zero, but not high enough to create the irreversible poration and ablation. Although the region of cortex is ablated, meaning the neurons have been destroyed, clearly no evidence of coagulation or thermal events can be seen in any of the tissue slices. Around 70% of the neurons in the neocortex are eliminated in the first 200 μm with a decrease in the number of ablated cell with depth (Supplementary Figure 4).

**FIGURE 4:**
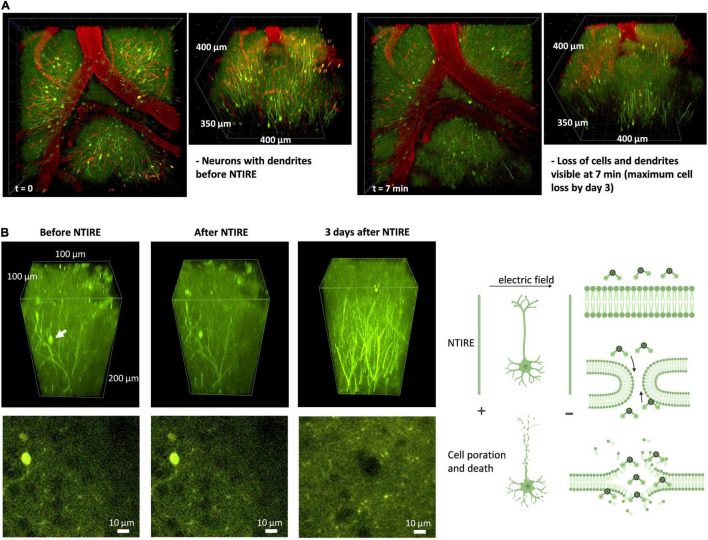
Principal of NTIRE: Ablation timeline for individual neurons and new projections. **(A)** Initial cell death and destruction of dendrites is seen in the initial minutes. Unlike the thermal ablation which destroys vessels and neurons immediately and leaves the brain with a coagulated region, NTIRE does not show complete cell loss until day 3 (corresponding well with histology) and shows numerous signs of allowing the ablated brain region to recovery from the ablation to some degree. **(B)** We follow the time course of an example neuron (white arrow) in a section of cortex which has been ablated with NTIRE using the microelectrodes (top panels) and a slice at the depth of the neuron (bottom panels). The neuron before and 10 min after NTIRE shows no specific change, although the irreversible electroporation event has taken place, and the neuron is on a trajectory toward cell death. Interesting the majority of neurons will be cleared away by day 3, corresponding well with histology (Supplementary Figure 4). Arrival of immune cells, Dendritic Cells seen here, at days 3 and 4, in particular seemingly concentrating themselves outside of vessels. By day 7 the peak concentration of Dendritic Cells is reached. Before and immediately after NTIRE, no change is seen (Supplementary Figure 5).

Unlike the thermal ablation which destroys vessels and neurons immediately and leaves the brain with a coagulated region, NTIRE does not show a complete cell loss right after the stimulation (corresponding well with histology). However, already at 7 min, a cell and dendrite loss already started (Figure 4A). The panel B, shows a section of cortex highlighting an individual pair of neurons in the field between the two microelectrodes. Before (top left panel) and several minutes after (middle panel) the NTIRE event is performed, the neurons are clearly visible. Indeed, neurons can often be visible for more than 24 h after NTIRE. The peak clearance of neurons is at approximately day 3; any neurons visible at or after day 3 will not be removed. Interestingly, as seen in the Figure 4B (right panel) for the same section of cortex, although the neurons have died and been cleared away, enervation of fibers from a region below the ablated cortex can be seen. This is a very interesting result, as this is not the case in a thermally coagulated section of neural tissue, where there is induction of axonal sprouting and synchronous activity (Carmichael and Chesselet, 2002). This could indicate an accelerated time-line for recovery, compared to the case of a thermal ablation, avoiding also the possible generation of epilepsy following cortical injury (Prince et al., 2009). Day 3, and also day 4, corresponds to a definite and visible increase in dendritic cells, specifically around the vessels in a region which has experienced the NTIRE event (Supplementary Figure 5). Day 7 seems to be the peak of dendritic cell concentration. In general, however, due to the lack of a thermal coagulated necrosis of tissue, it seems that the immune response and recovery is much similar to a mild stroke, with recovery on a much faster time-scale compared to thermal ablation. This is likely related to the fact that vasculature and extracellular matrix is completely in place, and there is no sign of tissue scarring.

### Non-thermal Irreversible Electroporation Can Suppress Epileptiform Activities (Interictal Spikes)

**FIGURE 5:**
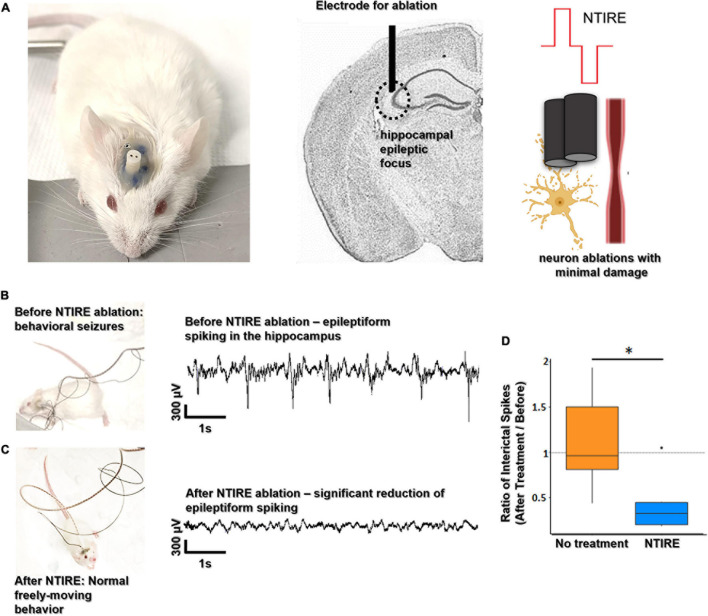
NTIRE ablation of hippocampal seizure foci. **(A)** A hippocampal seizure focus created via a standard kindling protocol (details in the section “Materials and Methods”) and an electrode is placed at the boarder of CA3. A two-electrode implant (100 μm electrode diameter, 50 μm spacing) used for the kindling protocol is then used for the treatment. **(B,C)** Video monitoring (representative of behavioral epileptic state available in Supplementary Material) and electrophysiological recordings in the hippocampus of animals prior to ablation show behavioral epileptic state and pathological epileptiform activity. Video monitoring and electrophysiological recordings in animals after ablation show no evidence of behavioral seizures and electrophysiological activity has a significant decrease in interictal epileptiform activity. **(D)** Evaluation of electrophysiological effects due to the NTIRE ablation (*n* = 12). The ratio (after treatment on before NTIRE) of epileptiform spikes during is dramatically reduced for the NTIRE mice (orange, *n* = 6) compared to the no treatment group (purple, *n* = 6), and no further behavioral seizures were observed. Wilcoxon test as been performed (*p-value = 0.02597).

Figure 5 shows the results of the NTIRE ablation applied to epileptic foci in the mouse hippocampus. As seen in Figure 5A, a two-electrode implant is placed at the border of CA3 in the mouse hippocampus. Animals had previously been rendered epileptic with a standard kindling protocol at this location (detail in the Supplementary Material). Animals under continuous video and electrophysiological supervision displayed behavioral epileptic state and pathological electrophysiological activity. As shown in Figure 5B, all the animals were kindled and were having burst of interictal spikes. For the ones treated with an NTIRE ablation no longer displayed behavioral seizures and no longer showed pathological electrophysiological activity (representative of behavioral epileptic state available in Supplementary Material). The ablation rendering the animal seizure-free is important, but the lack of coagulated tissue in the NTIRE ablation has perhaps additional and positive consequences regarding recovery after ablation. In order to demonstrate this impact on hippocampal oscillation, a ratio of the detected interictal spikes (spikes detection after treatment/spikes detection before treatment) as been performed. The No treatment group has a ratio ∼1 meaning that there is no decrease of the number of spikes detection with time. But NTIRE ablation induced a significative reduction of the interictal spiking (*p* value = 0.02597) is a technique of ablation that can also be performed at depth like in an epileptic focus located in the hippocampus, similar cell death is seen with a corresponding reduced tissue damage and lack of thermal coagulation (Supplementary Figure 1C).

## Discussion

Non-thermal irreversible electroporation is a well-established method for the ablation of many types of tissue and tumors (Garcia et al., 2010, 2011, p. 2; Ellis et al., 2011; Hjouj et al., 2012). The phenomenon of irreversible electroporation occurs when an applied electric field in the targeted tissue region is above a critical value, typically taken between 1,500 V/cm and 2,500 V/cm (Yarmolenko et al., 2011; Savic et al., 2016). Usually, the electric field needed to achieve irreversible electroporation is associated with significant tissue heating and thermal damage. However, Davalos et al. (2005) showed that irreversible electroporation can be generated without thermal damage by applying certain pulse parameters, and that NTIRE can be used to destroy considerable volumes of tissue *in vivo* without heating (Rodriguez et al., 2015). Indeed, researchers have shown the use of NTIRE in small and large animal models in the prostate, (Hjouj et al., 2012) liver (Garcia et al., 2011) and on implanted mouse sarcomas (Ellis et al., 2011). Studies outside the CNS have shown that NTIRE preserves the extracellular matrix and major blood vessels. In NTIRE studies outside the CNS, ablation is fast to apply and fast to resolve, permitting early repopulation of the ablated zone with healthy cells (Joshi and Schoenbach, 2002; Hjouj et al., 2012).

This work here describes the first time an NTIRE procedure has been coupled to live two-photon imaging in a mouse model, to follow its effects as they evolve. Focal lesions were created in the right somatosensory and parietal cortex of the mouse, and subsequently in the hippocampus to destroy seizure foci. Our results have shown that very short pulses durations per period, and correspondingly low RMS-voltages and low powers, cause only vasoconstriction of the vessels while destroying neurons. This finding indicates that NTIRE could be used safely in the brain to ablate pathophysiological structures while blood vasculature is maintained, in significant contrast to thermal ablation methods (Rossmeisl et al., 2013). We did not observe large-scale edema and swelling following the NTIRE ablations, in contrast to findings following thermal ablation with TIRE. The method may present a distinct new tool in epilepsy as a new option to treat seizure foci, rather than complete resective surgery or present thermal ablation techniques such as radiofrequency or LITT.

Our NTIRE ablations produce a sharply delineated volume of ablated tissue making it, as we have shown, suitable for treatment of epileptic foci in deep structures. The negligible heat generation during the ablation leaves extracellular matrix and major blood vessels undamaged. As described in this paper, we have shown several advantages over thermal ablation techniques related to bleeding and vasculature, but a second and very interesting finding is that, since NTIRE ablates tissue non-thermally rather than causing cell death through coagulative necrosis (as in thermal methods such as RF and laser ablation), it may accelerate the tissue recovery and regeneration process. The possibility of tissue to regenerate within the ablated epileptic foci, in part certainly due to the undamaged vasculature and lack of scarring, must be studied in detail in longer term experiments. However, our preliminary results show the recruitment of immune system cells, much more like brain recovery from mild stroke rather than a full thermal coagulative necrosis.

## Conclusion

The results of our experiments indicate that the advantages of NTIRE over thermal ablation technologies in the brain are significant. The improved safety profile of the NTIRE method in epilepsy should accelerate clinical exploration and translation. Indeed, our preliminary results are the first step toward using NTIRE as a focal ablation treatment clinically in epilepsy. Our results are perfectly in line with the results of previous studies that have shown that NTIRE, in tissue outside the brain, has many beneficial properties compared to thermal ablations, including better regeneration of tissue in the ablated zone and correspondingly improved avoidance of scar formation (Davalos et al., 2005). Currently, NTIRE is used in patients with tumors in organs such as the kidney, pancreas, lungs, and prostate (Rodriguez et al., 2015; Savic et al., 2016). An additional benefit, which might be ideal for epilepsy, is that the method allows work in tissue regions and organs with high densities of blood vessels, such as the insula of the brain, due to the insignificant impact on vascularization (Maor et al., 2007). Compared to thermocoagulation, the method completely avoids emboli and thrombosis after treatment (Davalos and Rubinsky, 2008). Even in cases of extremely high exposure to NTIRE, specific cases where clinicians want to electroporate tumor cells to allow the injection of chemotherapies, the BBB remains disrupted for 3 days post treatment and is reversed by 8 days (Hjouj et al., 2012). We hope that the results presented here stimulate further research into the NTIRE method as a significant alternative to thermal processes in the focal ablation process currently used to treat numerous epilepsies.

## Data Availability Statement

The raw data supporting the conclusions of this article will be made available by the authors, without undue reservation.

## Ethics Statement

The animal study was reviewed and approved by the National Animal Studies Committee of France (authorization no. APAFIS20359-2019041816357133v5).

## Author Contributions

AW conceived the project and wrote the manuscript with input from the other authors, including MC, IV, RG, RC, VJ, AT, and FB. EA, SS, and PW performed the experiments. BB performed finite-element simulations. EA and FM analyzed the neural data. All authors contributed to the article and approved the submitted version.

## Conflict of Interest

The authors declare that the research was conducted in the absence of any commercial or financial relationships that could be construed as a potential conflict of interest.

## Publisher’s Note

All claims expressed in this article are solely those of the authors and do not necessarily represent those of their affiliated organizations, or those of the publisher, the editors and the reviewers. Any product that may be evaluated in this article, or claim that may be made by its manufacturer, is not guaranteed or endorsed by the publisher.
