# Supplementary material for: Non-thermal Electroporation Ablation of Epileptogenic Zones Stops Seizures in Mice While Providing Reduced Vascular Damage and Accelerated Tissue Recovery
Source: Front Behav Neurosci. 2021 Dec 24;15:774999. doi: 10.3389/fnbeh.2021.774999 (PMC8740210; doi:10.3389/fnbeh.2021.774999)
Supplement: Supplementary file 1 [file Table_1.DOCX]

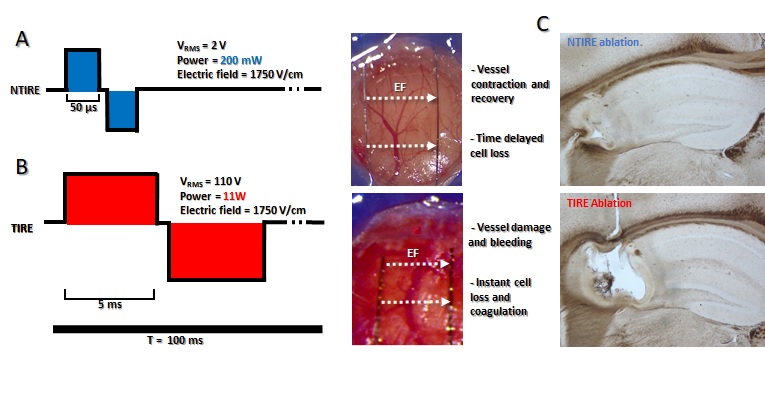


**Figure S1: Main Differences between a NTIRE and TIRE ablation** An NTIRE ablation is created by a pulse with an RMS voltage low enough to have an associated power which causes no thermal heating (in this figure 2 V_RMS_ and only 200 mW). However, the instantaneous electric field must be high enough to create an irreversible electroporation of neural membranes (in this figure 1750 V/cm, well above an estimated threshold of 1000 V/cm). As seen in the image, bleeding is not visible. B. A TIRE or traditional thermal ablation simply utilizes an RMS voltage which delivers a power high enough to cause coagulation. In this example, we maintain the same electric field (EF, white dashed arrows), but utilize 11W. As seen in the image the bleeding can be extensive, making any long-term analysis via the two-photon not possible. C. Either an NTIRE ablation (non-thermal) or a TIRE ablation (thermal) is performed. Example of histological evaluation of the hippocampus in the NTIRE vs TIRE showings markedly significantly increased tissue damage and evidence of thermal coagulation in mice treated with the thermal ablation. In both cases however, the epileptic structure has been ablated.


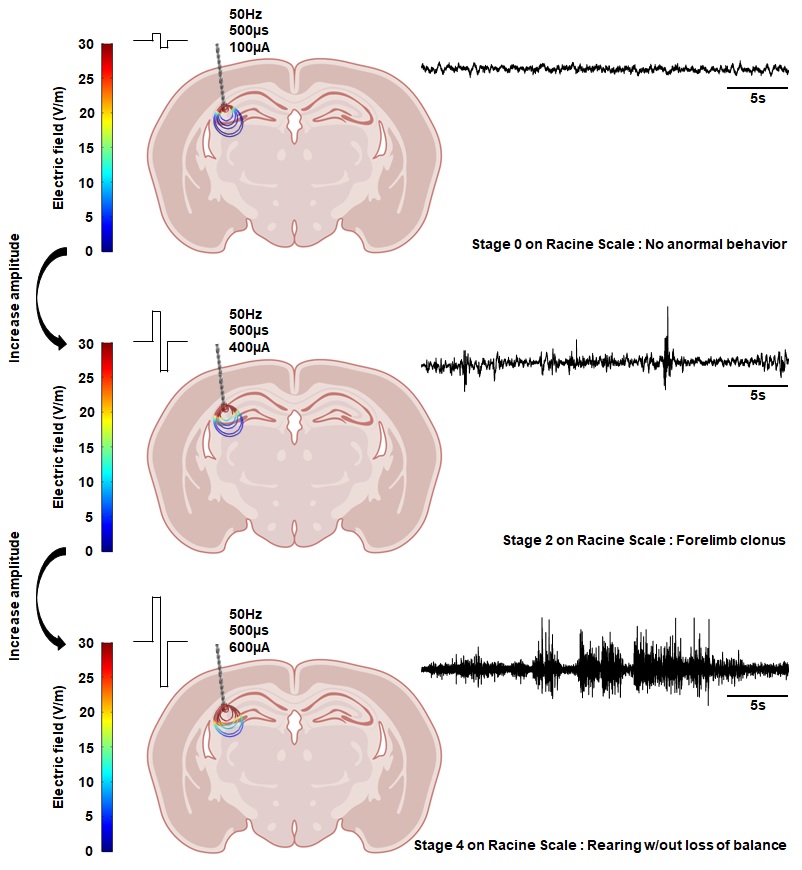


**Figure S2. Epileptic state method of induction.** A twisted pair electrod is implanted in the hippocampus and the current appleid is increased in order to evoke a stage 4 on Racine Scale. When this threshold is find for each mice, this stimulation is repeated 10 times in one hour to evoke an epileptic state.


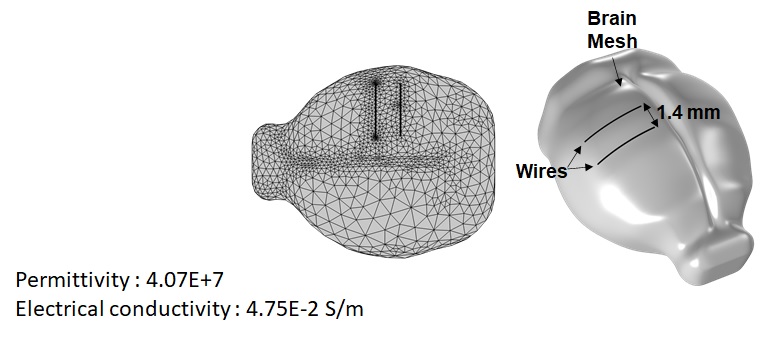


**Figure S3. Finite Element Model :** A custom mouse brain mesh model was designed on Blender software and then imported in COMSOL Multiphysics 5.5. The distance and the voltage applied between the electrodes were taken in account to recreate as closely as possible the experimental conditions of the Cortical NTIRE ablation, 245 V between ≃ 1.4 mm


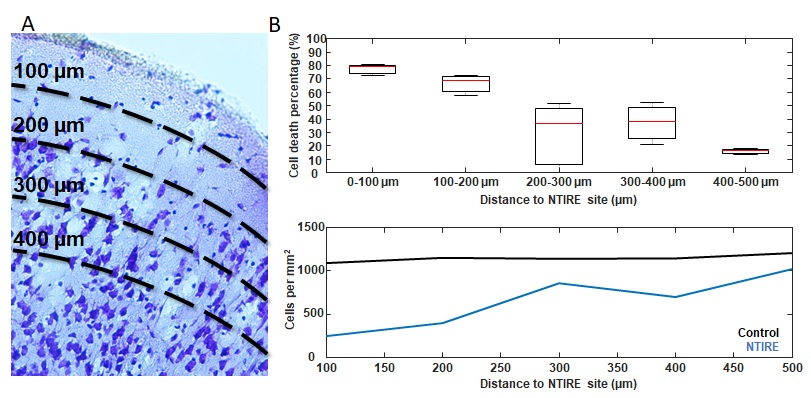


Figure S4. Cell death in function to the distance, 4 days after the NTIRE stimulation. **A.** Neurons identified were counted via ImageJ depending on their distance from the cortex. **B.** Cell death decrease in function of the distance from the cortex. Around 70% of the neurons disapear in the first 200µm. In the same way, cell density is then increased and regain a normal amount of cell after 500µm in depth.


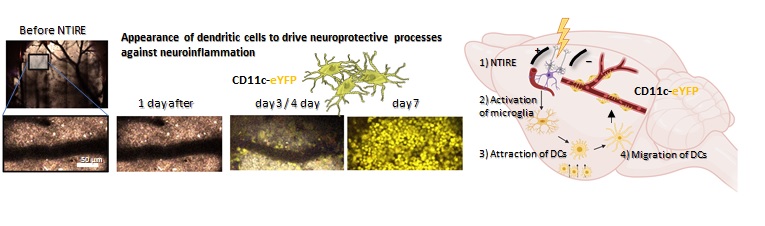


**Figure S5. Recruitment of immune cells.** Arrival of immune cells, Dendritic Cells seen here, at days 3 and 4, in particular seemingly concentrating themselves outside of vessels. By day 7 the peak concentration of Dendritic Cells is reached. Before and immediately after NTIRE, no change is seen.
